# One Raman DTS Interrogator Channel Supports a Dual Separate Path to Realize Spatial Duplexing

**DOI:** 10.3390/s24165277

**Published:** 2024-08-15

**Authors:** Cheng-Kai Yao, Chun-Hsiang Peng, Hung-Ming Chen, Wen-Yang Hsu, Tzu-Chiao Lin, Yibeltal Chanie Manie, Peng-Chun Peng

**Affiliations:** 1 Department of Electro-Optical Engineering, National Taipei University of Technology, Taipei 10608, Taiwan; t109658093@ntut.org.tw (C.-K.Y.); t111658059@ntut.org.tw (C.-H.P.); yibeshmamaru@gmail.com (Y.C.M.); 2 Marine Geology and Energy Department, Industrial Technology Research Institute, Tainan 71101, Taiwan; farmerchen@itri.org.tw (H.-M.C.); wyhsu26@itri.org.tw (W.-Y.H.); 3 Department of Electrical Engineering, National Taipei University of Technology, Taipei 10608, Taiwan; tclin@ntut.edu.tw

**Keywords:** Raman-distributed temperature sensing, spatial duplex measurement, free space optics

## Abstract

Deploying distributed fiber-optic sensor (DFOS) technology to gather environmental parameters over expansive areas is an essential monitoring strategy in the context of comprehensive searches for anomalous places. This study utilizes a single temperature measurement channel within a commercial Raman-based distributed temperature sensing (RDTS) interrogator and divides it into two separate, uncorrelated paths to enable spatial duplex temperature measurements. The distinction between temperature events corresponding to each path in the dual separate path (DSP) in RDTS can be achieved when temperature events are concurrently occurring in the DSP. Additionally, the RDTS–DSP solution may integrate free space optics (FSO) into its fiber path, which serves to enhance the user-friendliness, scalability, and cost-effectiveness of DFOS technology. An RDTS measurement channel can effectively function as a DSP, thus doubling the RDTS measurement pathway, and can be combined with FSO to significantly improve RDTS performance.

## 1. Introduction

The advancement of fiber-optic sensing technology has been an evolutionary process spanning numerous years. Fiber-optic sensors offer a host of advantages, including their compact dimensions, high sensitivity, resistance to electromagnetic interference, and versatility, establishing a strong foundation for their pervasive application in diverse sensing domains [1,2,3,4,5]. Despite these inherent benefits and superior performance relative to other types of sensors, the widespread adoption of fiber-optic sensors in practical scenarios has been hindered by the initial costs associated with ancillary equipment, optical cable installation, and sensor packaging [6]. To drive increased adoption, efforts must be directed toward enhancing the functionality and affordability of fiber-optic sensors. Notably, the optical signal transceiver is the sole component of the fiber-optic sensor that necessitates energy, with the fiber itself operating without power over sensing distances extending beyond several tens of kilometers. This inherent characteristic underscores the energy efficiency and potential carbon footprint reduction afforded by fiber-optic sensors, underscoring their pivotal role in future applications.

Fiber-optic sensors can be broadly classified into three categories: those that rely on fiber Bragg grating (FBG) configurations [7,8,9,10,11,12,13,14], those that depend on fiber-optic interferometer configurations [15,16,17,18,19,20,21,22], and those that use DFOSs based on Rayleigh, Raman, or Brillouin scattering effects [23,24,25,26,27,28,29,30,31]. The exceptional sensing sensitivity of fiber-optic sensors based on interferometers to a wide variety of measurements is certainly noteworthy [15,16,17,18,19,20,21,22]; nonetheless, the majority of fiber-optic interferometer sensors are restricted to single-site observations. Compared to fiber-optic interferometer sensors, FBG-based sensors may not be as sensitive, but they can be cascaded and multiplexed to form multiple points of measurement [32,33,34,35,36], thus providing a broader measurement range. Comparable to FBG in sensitivity, DFOS’s ability to monitor the environment at all places along the fiber path, as opposed to only certain spots [23,24,25,26,27,28,29,30,31], significantly increases the usefulness of fiber sensing applications. However, when compared for similar utilities, commercial DFOS interrogators are typically more than twice as expensive as commercial FBG interrogators, which greatly discourages the idea of using DFOS interrogators for measurement work. Moreover, issues arise with the installation and upkeep of the fiber-optic cables. Therefore, it is imperative to improve the functionality and economic viability of DFOSs.

This paper demonstrates the utilization of a single temperature measurement channel from a commercially available RDTS interrogator to support temperature measurement in a DSP, where a fiber-optic coupler serves as a critical barrel hoop between the one temperature measurement channel from the RDTS interrogator and the two distinct temperature measurement paths. Furthermore, involving FSO in DSP temperature measurement can streamline the optical cable laying procedure, thereby reducing associated installation and maintenance costs [32,33,34,35,36]. This is particularly helpful in applications with obstacles due to natural topography. Additionally, the DSP employs two distinct reflected power signals to correspond to individual pathways, or one of the DSP’s routes creates a sub-DSP to discriminate in which sensing path temperature hot spots occur. In this RDTS temperature measurement project, the combined effect of the temperature detection path through the DSP, coupled with the flexibility of FSO technology, is anticipated to make RDTS more approachable for applications that necessitate temperature or related parameter monitoring across a wide area.

## 2. RDTS and Overview

**Figure 1:**
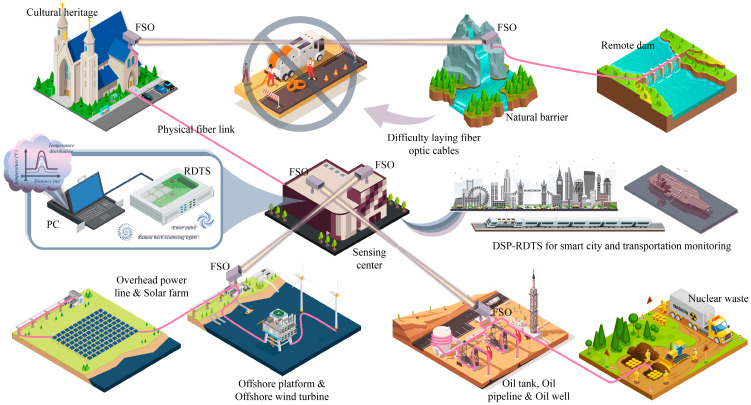
A vision of one RDTS interrogation channel to support the DSP in realizing spatial duplexing measurements.

Real-time temperature variation information can be obtained accurately and reliably using RDTS, which is essential in various fields such as industrial processes, environmental research, safety applications, and other temperature monitoring applications. This capability aids in enhancing safety precautions, raising awareness of the surrounding environment, and improving the operational efficiency of the item being tested. The monitoring applications related to temperature measurement using RDTS based on DSP architecture are portrayed schematically in Figure 1, which also incorporates the integration of the FSO application. A commercial RDTS interrogator in the sensing unit at the sensing center sends a pulse to the DSP or a DSP with an FSO link. Note that typical commercial RDTS interrogators contain two temperature measurement channels by default; however, these channels cannot be utilized simultaneously in parallel, and the other channel is often exclusively reserved for the optional purpose of temperature calibration [37]. Although there are RDTS products with more than two measurement channels, they come at a higher cost. Typically, RDTS transmits pulses at 1064 nm or 1550 nm, with their corresponding stimulated Stokes and anti-Stokes wavelengths being approximately ±40 nm and ±100 nm, respectively. The anti-Stokes backscattering light power of RDTS is more susceptible to temperature changes than the Stokes backscattering light power, which can serve as a reference element for temperature interrogation to compensate for the different loss coefficients during temperature demodulation [24,29]. The backward Stokes and anti-Stokes Raman scattering signals obtained from different locations along the fiber are directed to their corresponding avalanche photodetectors and data acquisition cards, and then to the personal computer (PC) for the presentation of temperature distribution information along the whole fiber path [23,29]. The RDTS used in this work is based on a 1064 nm pulse with a spatial resolution of 1 m and a sampling interval of 15 cm.

Widening the scope of fiber-optic deployment through the use of DSP–RDTS measuring technology enables more comprehensive implementation of smart city monitoring, including building health, traffic safety, and weather status. The monitoring of subsea cables, offshore wind turbines, offshore platforms, substations, overhead power lines, solar panels, oil-related industries, nuclear-related safety, the preservation of significant cultural artifacts, and other applications could all greatly benefit from the adoption of RDTS equipment. As shown in previous reviews [24,25], RDTS has demonstrated significant applications in temperature measurement for dam safety, power transformers, high-voltage cables, the oil industry, low-temperature environments, tunnel safety, hydrological studies, and climate surveys. Furthermore, the feasibility and advantages of combining FSO with fiber-optic sensing have been demonstrated in previous studies [30]; as a result, the combination of FSO and DSP–RDTS will yield more benefits. FSO technology is especially valuable in rugged or remote locations where installing fiber-optic cables is challenging, making it particularly useful for hydrological and geological surveys [32,33,34,35,36]. The characteristics of RDTS and its application value are very much in conformity with the objectives of green energy and sustainable development, and with the backing of DSP and FSO technologies, RDTS can be leveraged in additional applications to maximize its potential value.

## 3. Experiments and Discussions

### 3.1. DSP–RDTS Scheme

**Figure 2:**
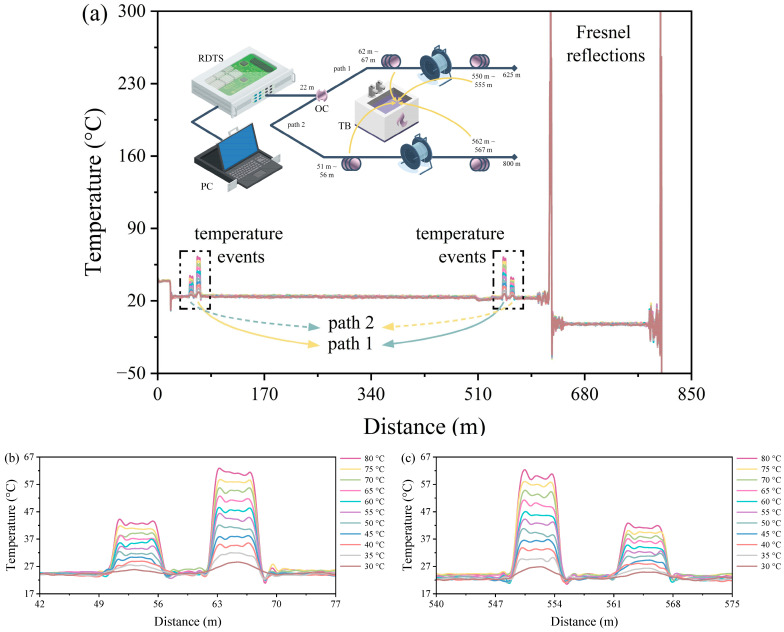
(**a**) Architecture diagram and temperature distribution measurement of DSP–RDTS. (**b**) Front section zoomed-in temperature distribution of the optical fiber path. (**c**) Back section zoomed-in temperature distribution of the optical fiber path.

Figure 2a demonstrates the experimental DSP–RDTS scheme and the temperature distribution plots. The DSP architecture is established using a 60:40 power ratio fiber-optic coupler (OC) at a distance of 22 m to split a single temperature monitoring channel in the RDTS interrogator into two separate pathways. This results in pulses with varying power being transmitted to two separate paths. The lengths of paths 1 and 2 in the DSP are 625 m and 800 m, respectively. Specific segments of the optical fibers in path 1 (approximately 62 m to 67 m and 550 m to 555 m) and path 2 (around 51 m to 56 m and 562 m to 567 m) are submerged in a thermostatic bath (TB) to measure the temperatures at various water temperatures. The temperature distribution graph indicates a noticeable temperature change at 22 m, attributed to the power variation arising from the connection to the OC. As a result, the temperature distribution baseline is adjusted after 22 m using the built-in Pt100 of the RDTS interrogator to bring it to ambient temperature. Additionally, a sharp peak is observed in the temperature distribution due to Fresnel reflection caused by the surface at the end of the fiber path [30]. Furthermore, slight temperature fluctuations can be seen at around 510 m, likely stemming from minor imperfections near the fusion point of the fiber. The large decrease in temperature distribution after 625 m is because path 1 is only long enough to reach about 625 m, and the temperature distribution is calculated by superposing the reflected signals from the measurement paths of both path 1 and path 2.

Figure 2b,c depict the enlarged temperature distributions of the optical fiber test sections at the front and rear segments of paths 1 and 2 in Figure 2a. The temperature is measured by heating the water from 30 °C to 80 °C at 5 °C intervals with the data collection. Path 1, the path with the higher temperature distribution reading, is the one with 60% of the 60:40 power splitting ratio, and path 2, on the other hand, with a lower temperature distribution reading, is the one with 40% of the 60:40 power splitting ratio. Moreover, due to the OC splitting and the fact that the positions of the fiber-optic test sections of the two paths do not overlap, the measured temperature will be smaller than the actual temperature due to the attenuation of the reflected signals. That being said, this is a normal phenomenon; even if there is a variance from the actual temperature, the temperature distribution result still reflects the temperature change and regular change. Additionally, by embracing this OC with optical power imbalance, in some cases, it will simply identify which path the temperature event is occurring in. For example, if the temperature event in path 1 is above 70 °C, the temperature event in path 2 may surpass 100 °C if it follows the same temperature distribution level as path 1. Thus, by knowing where the temperature conditions of the environment to be tested will fall, it is possible to determine the path of a temperature event. For instance, RDTS is essential for temperature monitoring of subsea cables [29]; in the event of a cable without failure, the temperature underneath the sand where the cable is located is between 50 °C and 70 °C [38], and in the event of cable failure, the temperature of the cable rises by about 4 °C [39], so if the temperature distribution level exceeds 4 °C higher than the upper limit of path 2, it is possible to tell that a temperature event must have occurred in path 1. Of course, this is just an example, and since the effect of heat conduction in the fiber-optic cable is ignored, there may be some discrepancies between the temperatures and the results of the field measurements. In order to quantify the results of temperature distribution measurements, the center of the temperature change resulting from the temperature event serves as the sampling point for data quantification.

**Figure 3:**
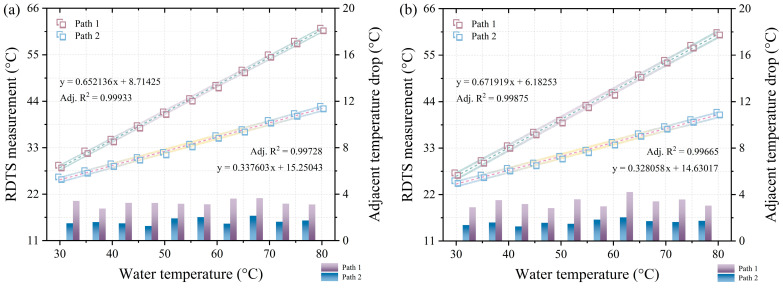
(**a**) Quantitative results of the temperature distribution in Figure 2b. (**b**) quantitative results of the temperature distribution in Figure 2c.

The quantification results shown in Figure 2b,c are represented in Figure 3a,b, respectively. A temperature distribution error, characterized by an S-shaped pattern occurring during sudden temperature changes, leads to the accurate measurement of only close to the central point of the temperature distribution when the measured temperature is close to room temperature, given a spatial resolution of 1 m. This explains why only the center of the temperature distribution is used for data quantification. The corresponding data quantification points in Figure 3a are situated at 53.55 m and 65.1 m in Figure 2b, while those in Figure 3b are at 552 m and 564.45 m in Figure 2c. The left vertical axes of Figure 3a,b portray the RDTS measurements from 30 °C to 80 °C for both path 1 and path 2, revealing that the slopes of the linear fit for path 1 are greater than those for path 2. The white and gradient-colored areas surrounding the linear fit’s dotted line represent the 95% confidence band and 95% prediction band, respectively. The right vertical axis displays the data portion of the bar graph, representing the difference between two adjacent temperature measurements in paths 1 and 2. For instance, the drop between the temperatures measured at 35 °C and 30 °C. The average adjacent temperature drops for the front and rear sections of path 1 and path 2 in the data section of the long bar are 3.246 °C, 3.322 °C, and 1.673 °C, 1.622 °C, respectively. From Figure 3a,b, it is apparent that the temperature distribution in the front and back parts of the fiber under testing for either path 1 or path 2 is similar, validating the viability of DSP measurement.

### 3.2. DSP–RDTS with FSO Scheme

**Figure 4:**
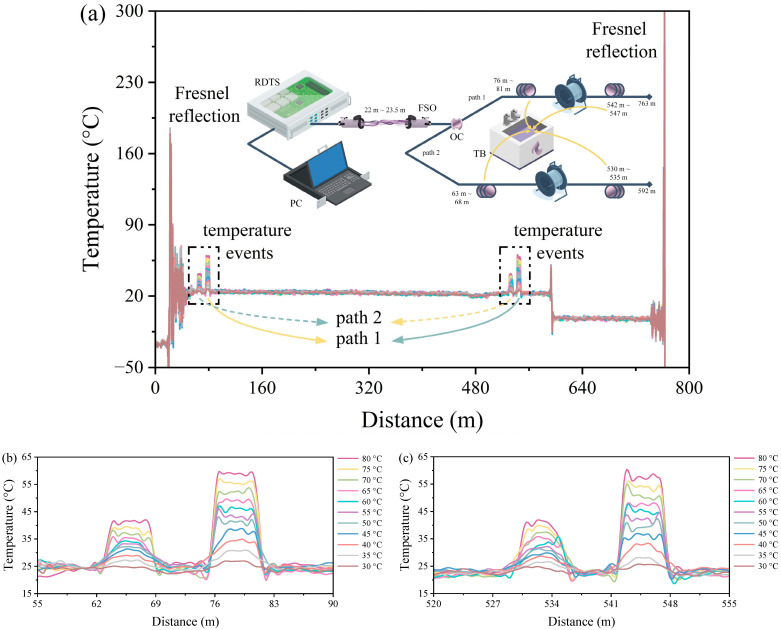
(**a**) Architecture diagram and temperature distribution measurement of DSP–RDTS with FSO. (**b**) Front section zoomed-in temperature distribution of the optical fiber path. (**c**) Back section zoomed-in temperature distribution of the optical fiber path.

**Figure 5:**
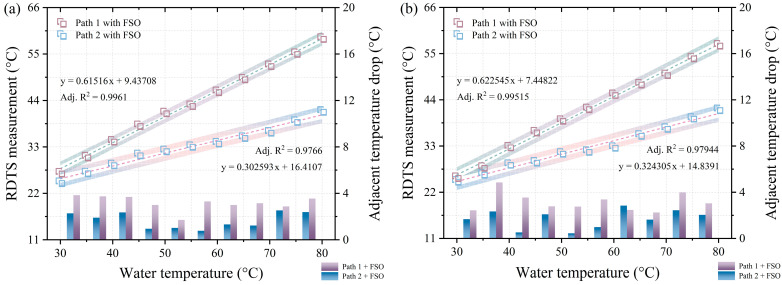
(**a**) Quantitative results of the temperature distribution in Figure 4b. (**b**) quantitative results of the temperature distribution in Figure 4c.

The experimental design and temperature distribution of the DSP–RDTS scheme with FSO involvement are displayed in Figure 4a. The 1.5 m FSO link is set up at 22 m, the 60:40 OC is immediately next to the FSO link, and there is also Fresnel reflection and temperature ringing appearing in the temperature distribution near the FSO link due to the presence of an air section. The endpoints of paths 1 and 2 are 763 m and 592 m from the measurement start point of the RDTS interrogator, respectively. The fiber-optic test sections are situated in paths 1 (76 m to 81 m and 542 m to 547 m) and 2 (63 m to 68 m and 530 m to 535 m), as shown in Figure 4b,c. The quantitative results of the temperature distributions in Figure 4b,c are presented in Figure 5a,b, respectively, which are quantified as previously mentioned. It can be observed that there are some temperature fluctuations in the temperature distribution of the optical fiber test section in Figure 4b,c, which are caused by the introduction of FSO to the optical fiber path, which will increase optical loss and interfere with temperature measurements. However, it is interesting to note that in the previous RDTS temperature measurements [30], where only a single fiber path was used, the inclusion of FSO did not have much impact on the temperature measurements. Consequently, the weakening of the accuracy of the temperature distribution is not directly caused by the FSO link, but rather by the fact that the use of the OC itself causes a power loss of more than half of the original pulse energy emitted by the RDTS interrogator at the beginning of paths 1 and 2, so if this loss is combined with the loss in the FSO link (roughly 20% of the original pulse energy), then this cumulative loss reaches a threshold that may affect the temperature measured accuracy by the RDTS interrogator. Although the loss of optical power due to the OC and FSO can affect measurement accuracy, this can be overcome by increasing the pulse power emitted by the RDTS interrogator. Moreover, it is certain that the temperature distribution still varies regularly following the water temperature. The quantification findings shown in Figure 5a,b show that, as a result of the decrease in temperature precision, the 95% confidence band (white area) and 95% prediction band (gradient-colored area) are bigger in comparison to the scenario without FSO. The quantified mean adjacent temperature drops for paths 1 and 2 are 3.191 °C, 3.153 °C, and 1.689 °C, 1.704 °C, respectively. These results are comparable to the DSP scheme without FSO, demonstrating that despite FSO involvement adding extra losses and consequently causing fluctuations in the temperature distribution, the FSO-equipped DSP scheme does not substantially affect the temperature measurement levels at different water temperatures. Conclusively, the DSP measuring technique, which involves FSO, remains capable of detecting changes in water temperature by analyzing variations in the temperature distribution of the measurement.

### 3.3. DSP–RDTS with FSO within One Path Scheme

**Figure 6:**
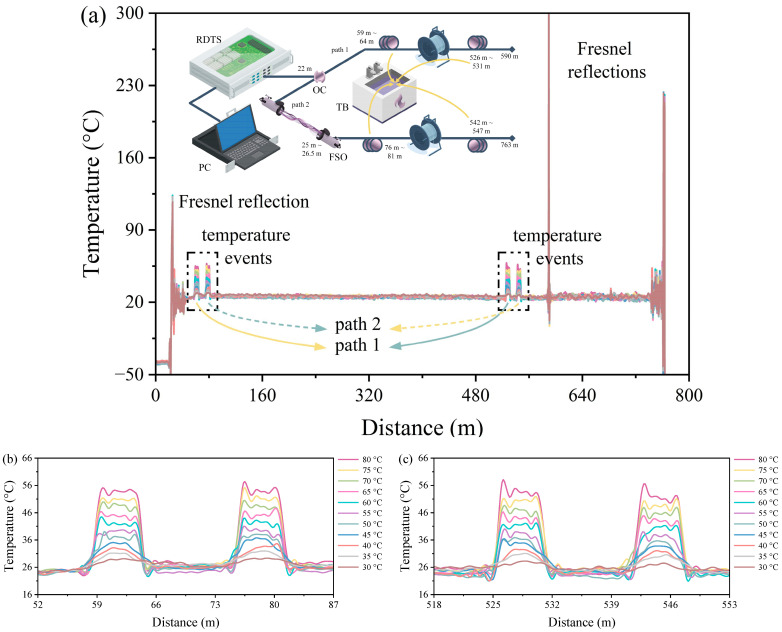
(**a**) Architecture diagram and temperature distribution measurement of DSP–RDTS with FSO. (**b**) Front section zoomed-in temperature distribution of the optical fiber path within one path. (**c**) Back section zoomed-in temperature distribution of the optical fiber path.

**Figure 7:**
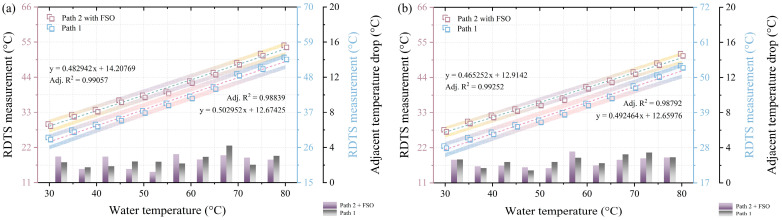
(**a**) Quantitative results of the temperature distribution in Figure 6b. (**b**) quantitative results of the temperature distribution in Figure 6c.

Figure 6a shows the experimental architecture and temperature distribution of the DSP–RDTS scheme with FSO installed in only one path. In path 1, via the 40% optical power ratio, the RDTS interrogator’s pulse does not pass, whereas in path 2, via the 60% optical power ratio, it passes via the OC and then passes through the FSO link. The fiber-optic test sections are located at 59 m to 64 m and 526 m to 531 m in path 1 (terminating at 590 m) and at 76 m to 81 m and 542 m to 547 m in path 2 (terminating at 763 m), respectively. Since the loss value of FSO is about 20% of the power of the pulse itself, if it is added to the loss of path 2 (about 50% of the power of the pulse itself), then it is just equal to the loss of path 1 (about 70% of the power of the pulse itself). Then, it can be seen from Figure 6b,c, namely the magnification of the temperature distribution of the optical fiber test section in Figure 6a, that the temperature distribution levels of paths 1 and 2 are similar. As was previously indicated, the extra loss due to FSO up to the threshold amount may have an impact on how well the RDTS interrogator measures temperature. Nonetheless, changes in temperature measurement precision were also noted in the path in which FSO was not involved. Please note that the RDTS interrogator will also use the reflected signal from the path containing the FSO link when interpreting the temperature of the path without FSO. As a result, the temperature distribution of the fiber under the testing section of the path without FSO will superpose with the temperature distribution of the non-testing fiber section (room temperature section) of the path with FSO, leading to this outcome. Figure 7a,b present the quantization results of the temperature distributions of Figure 6b,c, and the 95% confidence band and 95% prediction band are likewise larger than those of the DSP design without FSO. The quantified mean adjacent temperature drops for the front and back segments are 2.468 °C, 2.378 °C (path 2), and 2.509 °C, 2.519 °C (path 1), respectively. Given that the dual path of the DSP–RDTS scheme can function with FSO involvement, it is clear that changes in the temperature distribution can be used to detect changes in water temperature in the single path of the DSP–RDTS scheme with FSO participation.

### 3.4. DSP–RDTS with Sub-DSP within One Path Scheme

**Figure 8:**
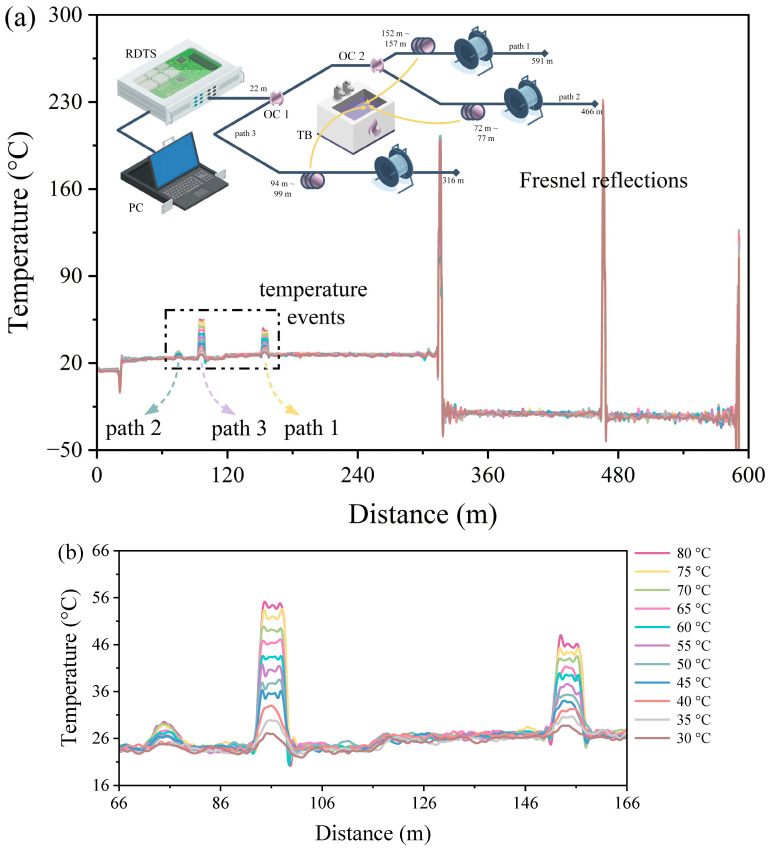
(**a**) Architecture diagram and temperature distribution measurement of DSP–RDTS with reference path. (**b**) Front section zoomed-in temperature distribution of the optical fiber path.

**Figure 9:**
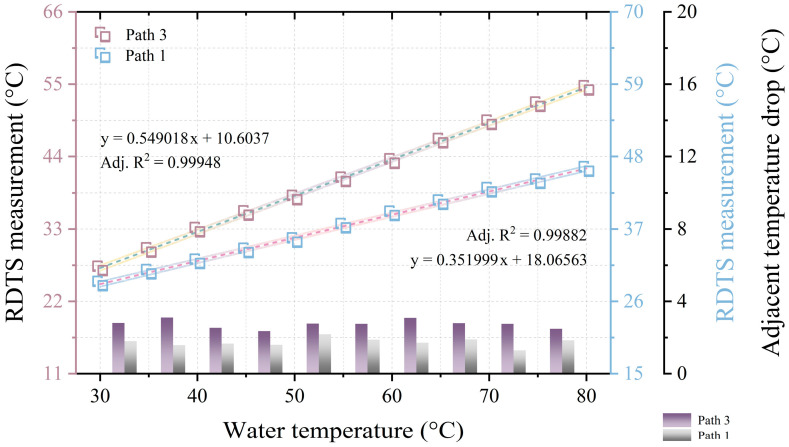
Quantitative results of the temperature distribution in Figure 8b.

As mentioned earlier, using the 60:40 OC in some specific temperature monitoring applications, it may be easy to know which path the corresponding temperature events are occurring in at different locations. To ascertain the paths along which temperature events occur, a more thorough methodology is required since temperature situations can change arbitrarily in some temperature measurement applications. Figure 8a shows an experimental arrangement in which another DSP is created on one of the DSP–RDTS paths, and the measured temperature distribution. A 60:40 optical power ratio also characterizes OC2, which is next to OC1. The portion of OC1 with a 40 optical power ratio is path 3, and the portion of OC1 with a 60 optical power ratio is then divided by OC2 into paths 1 (60% optical power ratio) and 2 (40% optical power ratio). The power splitting ratio of paths 1, 2, and 3 after computation is 39:15:46, which is close to the ratio of 36:22:42 of the maximum temperature distribution reading (80 °C) of the fiber test section in the zoomed-in temperature distribution in Figure 8b. Placed at 152 m to 157 m, 72 m to 77 m, and 94 m to 99 m, respectively, are the fiber-optic test portions for path 1, path 2, and path 3. Additionally, there is a deviation in the temperature level at 116 m in the temperature distribution, which was caused by a defect in the fusion point of the optical fiber. The primary concept of determining which path a temperature event occurs on is to assume that paths 1 and 2 are bundled together in parallel so that 80 m of optical fiber is placed in path 1 before it is bundled with path 2; then, when a temperature event occurs anywhere in the bundle of paths 1 and 2, the position of the temperature distribution in path 1 must be 80 m behind the position of the temperature distribution in path 2, as Figure 8b shows. Because of this, there is a strong likelihood that the temperature event will occur in path 1 as long as there are two temperature events in the temperature distribution that vary by 80 m and the temperature distribution is at the level of one big and one small. Figure 9 shows the quantitative results of the temperature distribution corresponding to Figure 8b, since path 2 is solely utilized as a reference path to determine on which path the temperature event occurs, and the reflected signal power of path 2 is small and is not used as the basis for the measurement of the temperature change. Interestingly, after using two OCs, the area of the 95% confidence band and 95% prediction band of path 1 can be compared with the DSP–RDTS architecture without FSO participation. It must be clarified that, in addition to the large drop in reflected power affecting the quality of temperature measurement by the RDTS interrogator, there is a difference in the wavelength traveling paths of Stokes and anti-Stokes signals when the light passes through the FSO link, and the power of the anti-Stokes signals passes through the FSO link more than the power of the Stokes signals, which also affects the quality of the temperature measurement. Stokes backscattering is not sensitive to temperature, but it is important for the quality of temperature measurements as a calibration basis for various loss coefficients. From the temperature distributions in Figure 4a and Figure 6a, it can be seen that the Stokes signal passes through the FSO link less because there is a marked increase in the temperature distribution at the FSO section, which means that the anti-Stokes signal passes through the FSO link more since the anti-Stokes power is directly proportional to the increase in temperature. Moreover, the quantified mean adjacent temperature drops for paths 1 and 3 are 1.741 °C and 2.742 °C, respectively.

**Figure 10:**
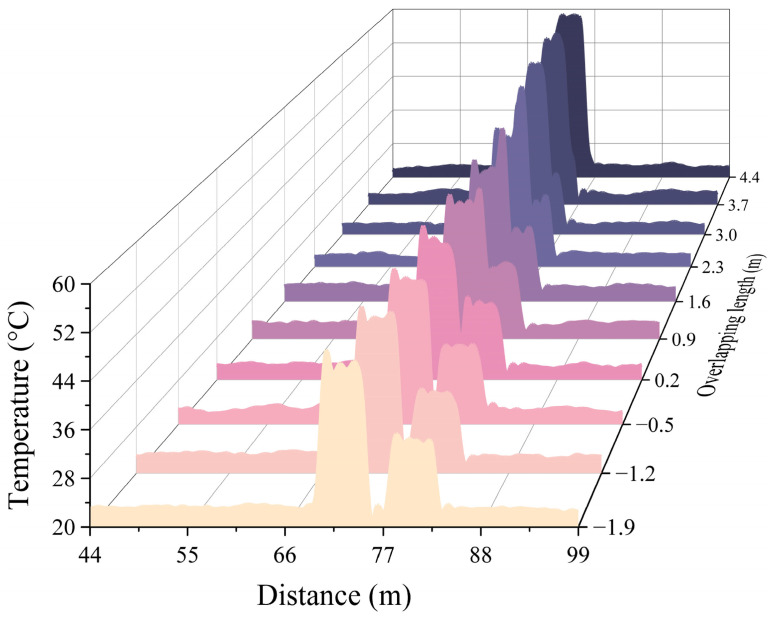
Overlap of temperature event positions for two separate paths in DSP-RDTS.

Nevertheless, when the temperature event location of path 3 overlaps with the temperature event location of path 1 or path 2, the previously discussed method of determining which path the temperature event happened on does not account for this circumstance. With this end in view, utilizing the DSP–RDTS design, Figure 10 displays the temperature distributions of the distinct paths when the locations of the temperature events coincide with one another. Specifically, it has been discovered that when temperature distributions coincide, either fully or partially, their temperature distributions will also increase as a result of superposition. This implies that even if path 3’s temperature event positions coincide with path 1’s or path 2’s, they can still be detected without having to take the issue of omission into account. Alternatively, it is possible that two temperature events occurring in path 3, 80 m apart and corresponding to the exact temperature profiles of paths 1 and 2, could be misinterpreted as occurring in path 1, although this is an extreme case and highly unlikely to occur. Furthermore, as previously indicated, raising the pulse power not only solves the issue of related losses leading to a decrease in temperature measurement accuracy but also theoretically permits upgrading to multiple separate temperature measurement paths instead of just the DSP architecture by connecting additional OCs. However, there is an upper limit to increasing the pulse power due to the upper limit of the received power of avalanche photodetectors. It is important to note that there are methods available to enhance measurement accuracy by reducing noise without increasing pulse power. One such technique is pulse coding [24,40,41]. Pulse coding involves modulating a laser in a specific pattern, allowing temperature information to be encoded in a way that is easier to separate from background noise. By using encoded pulses, the system can effectively differentiate signals from different locations along the fiber, thus improving the resolution and accuracy of temperature measurements. Additionally, the acoustic-optic modulator (AOM) enables precise control of the laser beam by quickly turning the light on and off or adjusting its intensity. This optimization of light-fiber interactions enhances Raman scattering signals. The AOM’s capability to change the light frequency helps to distinguish the Raman signal from other sources of noise, thereby improving the signal-to-noise ratio and allowing for better temperature measurements at lower power levels. By combining pulse coding with an acousto-optic modulator, the system can achieve a higher signal-to-noise ratio. Consequently, even with the same laser power, the system can detect smaller temperature variations more efficiently. This will address the issue of the upper limit of pulse power increase in multi-path measurements and the problem of low signal-to-noise ratio caused by power attenuation in multi-path measurements.

In the context of non-temperature events, the integrated Pt100 of the RDTS interrogator facilitates ambient temperature measurements and enables calibration of the ambient temperature level. In cases where the integrated Pt100 temperature sensor is positioned in one of the two paths, a discrepancy between the overall ambient temperature level measured by the RDTS and that of the integrated Pt100 temperature sensor suggests a change in the ambient temperature of the other path, thereby possibly indicating a change in its ambient temperature. Nevertheless, addressing scenarios with multiple paths and where ambient temperature changes are limited to specific path sections necessitates the use of additional arrangements of single-point temperature sensors to resolve this issue. Importantly, practical observations indicate that alterations in ambient temperature across the entire path do not notably impact the measurement of temperature events. For instance, if the temperature difference between the ambient temperature of one path and a temperature event in another path is 10 °C before the ambient temperature changes, the temperature difference will remain 10 °C even after the ambient temperature of that path changes. This constancy stems from the eventual superimposition of the dispersed energy from each measurement point along the different paths. Furthermore, the change in FSO path length causes related problems, such as the difference in wavelengths between the forward Stokes and anti-Stokes Raman scattering that causes the paths taken by the two wavelengths to be misaligned with the original paths they should have taken after passing through the FSO path. This may indirectly lead to different degrees of temperature distortion after demodulation depending on the FSO settings and path lengths, and a buffer path is needed to bring the forward Stokes and anti-Stokes Raman scattering back to the right track. Therefore, the impact of FSO path length on temperature measurement mainly depends on the optical design to minimize the chromatic aberration problem.

## 4. Conclusions

This study utilizes an orphaned temperature measurement channel in a commercial RDTS interrogator based on Raman technology, splitting it into two mutually unconnected paths, with each of its independent paths carrying out its temperature measurement work. It also enables the recognition of temperature events occurring along different paths. Moreover, the RDTS–DSP measurement scheme can use an FSO link as a substitute for fiber-optic links to improve the ease of use, scalability, and cost-effectiveness of DFOS technology. A measurement channel in the original RDTS interrogator can be converted into a DSP configuration by the OC, doubling the temperature probe measurement route of the RDTS interrogator along the entire fiber for spatial duplex temperature measurements; it can then be integrated with FSO, thus vastly enhancing the effectiveness of RDTS.

## Data Availability

The data presented in this study are available in this article.
